# Micro computed tomography for vascular exploration

**DOI:** 10.1186/2040-2384-2-7

**Published:** 2010-03-05

**Authors:** Lyubomir Zagorchev, Pierre Oses, Zhen W Zhuang, Karen Moodie, Mary Jo Mulligan-Kehoe, Michael Simons, Thierry Couffinhal

**Affiliations:** 1 Heart and Vascular Research Center, Dartmouth Medical School, Lebanon, NH, USA; 2 Cardiology Section, Dartmouth Medical School, Lebanon, NH, USA; 3 Radiology, Dartmouth Medical School, Lebanon, NH, USA; 4 Clinical Sites Research Program, Philips Research North America, Briarcliff Manor, NY, USA; 5 Inserm U828, Plateforme d'Innovation Biotechnologique de Xavier Arnozan, Université Victor Ségalen Bordeaux 2, Pessac, France; 6 Pôle Cardiothoracique, CHU de Bordeaux, Université Victor Ségalen Bordeaux 2, France; 7 Yale University School of Medicine, New Haven CT, USA; 8 Departments of Surgery, Vascular Section, Dartmouth Medical School, Lebanon, NH, USA

## Abstract

Vascular exploration of small animals requires imaging hardware with a very high spatial resolution, capable of differentiating large as well as small vessels, in both *in vivo *and *ex vivo *studies. Micro Computed Tomography (micro-CT) has emerged in recent years as the preferred modality for this purpose, providing high resolution 3D volumetric data suitable for analysis, quantification, validation, and visualization of results. The usefulness of micro-CT, however, can be adversely affected by a range of factors including physical animal preparation, numerical quantification, visualization of results, and quantification software with limited possibilities. Exacerbating these inherent difficulties is the lack of a unified standard for micro-CT imaging. Most micro-CT today is aimed at particular applications and the software tools needed for quantification, developed mainly by imaging hardware manufacturers, lack the level of detail needed to address more specific aims. This review highlights the capabilities of micro-CT for vascular exploration, describes the current state of imaging protocols, and offers guidelines and suggestions aimed at making micro-CT more accurate, replicable, and robust.

## Introduction

Micro Computed Tomography (micro-CT) presents unique opportunities for highly quantitative three dimensional imaging of models of disease implanted or induced in animal models. It is a structural imaging modality that can differentiate contrast-enhanced tissues or structures with high attenuation factors from non-enhanced soft tissues. Traditional use of micro-CT includes in vivo and ex vivo imaging applications such as screening for anatomical abnormalities and detection and quantification of anatomical changes in live animals or tissue samples removed from sacrificed animals. Micro-CT also plays an increasingly important role in the study of angiogenesis, a process that occurs naturally during development, tissue repair or abnormally in pathologic diseases.

Studying vascular development or the mechanisms of neovascularisation (angiogenesis, arteriogenesis or vasculogenesis) and evaluating the effects of pro or anti-angiogenic strategies require complete and accurate analysis of the neoformed vascular network. However, methods of assessment, such as histology with confocal or two-photon microscopy, laser Doppler, microangiography, fluorescent microspheres, magnetic resonance angiography, positron emission tomography, are not always precise or quantitative; they focus on a limited area of study, reveal capillary density primarily in 2 dimensions, and represent superficial blood flow (for details see review[1]). Currently, micro-CT is the only structural imaging modality that provides a high resolution volumetric representation of vascular structures that directly reflects the level of angiogenesis or inhibition/development of neo-vasculature. In combination with functional information from other imaging modalities such as fNMR, MicroPET, Ultrasound, or microscopy, micro-CT has the potential to advance the angiogenesis related research even further[2].

The spatial resolution of micro-CT volumes strictly depends on the X-ray source/detector geometry, which is dictated by the type of scanner. Currently, typical *in vivo *micro-CT scanners have resolutions ranging from 100 to 30 μm, while *ex vivo *scanners have resolutions from 30 to 1 μm.

In this review, mainly focused on micro-CT analysis of angiogenesis, we will describe standard protocols for animal preparation and the properties of contrast agents and vehicles to properly visualize vessels. The successive steps of image analysis, traps, and difficulties of quantification will be thoroughly detailed. Current limitations of micro-CT in vascular research will be addressed. We will also discuss other vascular applications and uses of micro-CT and perspective of its vascular application.

## Standard protocols for animal preparation

As with all imaging methods, animal positioning and preparation (pressure and or volume of infused contrast and solutions) should be as uniform as possible. In general, the animal is heparinized (100 IU/Kg ip) and deeply anesthetized (Ketamine/Xylazine 100 mg/kg/10 mg/kg). The animal is fixed into position on its back. When filling vessels for hindlimb ischemia or flank tumor analysis, the chest is opened and a cannula is sutured into the descending aorta with the tip of the needle facing the tail. The inferior vena cava is cut to allow infused solutions to exit the body. When filling the aorta for imaging of vasa vasorum, we have found that placing the cannula into the left ventricle and into the ascending aorta followed by a suture to hold this in place and prevent contrast from leading backwards works well. It may be helpful to notch the cannula circumferentially to hold the suture in place on the vessel. Filling of the heart vessels may be done by placing the cannula retrograde into the thoracic aorta. The contrast does not enter the left ventricle with an intact aortic valve, yielding a cleaner image. Saline solution (37°C, containing vasodilating agents such as adenosine and papaverin) is infused at 100 mmHg for 3 minutes to clear the specimen of blood and dilate the vessels to assist in maximum filling. At this point fixation with 2-4% paraformaldehyde may be desired. Fixation will allow for specimen preservation but may shrink vessels thus reducing filling of smaller vessels. The specimen is then ready for contrast infusion. Care should be taken to minimize bubbles in the fluid line as this may block filling of smaller vessels. For complete filling of vessels (arteries and veins), it is desirable to continue infusion of contrast past the point when it can be seen exiting the vena cava. If arterial filling alone is desired, it is necessary to administer a controlled volume of contrast agent and to use an agent that can be solidified rapidly when the agent has filled the desired vasculature. Also, it is important to limit the vessels that are damaged during animal preparation as the contrast will leak out of severed vessels changing the pressure and volume that are required for complete filling. Once the vasculature is filled and the contrast solidified, the specimen can be imaged immediately. Alternatively, the specimen may be placed in a suitable fixative (10% formalin, PFA Zinc, etc.).

## Contrast agents

Unlike bony anatomy, blood vessels provide very little inherent contrast for micro-CT imaging. The implementation of novel vascular contrast agents and vehicle has resulted in several new applications for micro-CT in the evaluation of micro vascular anatomy. Important functional and technical specifications of such contrast agents include high radio opacity, ease of manipulation and injection and physical properties adaptable to filling the desired vascular bed or organ.

Most clinical X-ray contrast agents are based on the element iodine. They are water soluble and therefore suitable for clinical radiography applications. Radiological contrast agents are water soluble solutions as well. One commonly used variety is based on a suspension of large insoluble particles of barium sulphate. The latter have better coating properties than the iodinated contrast media, and tend to form thin layers spread over the lining of tubes. However, it has been proven that insoluble particles tend to settle quickly and impair contrast homogeneity. This problem could be solved in part by using pulverized barium sulphate with 1 μm particles. Many other elements have higher atomic numbers than barium and can be used as contrast agents. Bismuth and lead (Pb) are good examples.

### Contrast agents for ex vivo imaging

Viscosity is one of the most important properties of the vehicle, because it influences contrast agent behavior and its applications for micro-CT (see Additional file 1 and table S1 for more details). The contrast agent must completely fill the vascular network to allow a precise study of the microcirculation: the actual resolution of vessels imaged with micro-CT is in the range of 5 to 20 μm (i.e., the effective voxel size). Depending on the contrast properties (especially viscosity), penetration of the vehicle through the vessel tree could be limited. For example, in an arterial injection, increased viscosity limits vehicle penetration at the arteriolar level only (capillaries are not filled). This aspect could be of great interest in arterial/venous delineation in micro-CT analysis, as the vehicle does not flow freely through the capillary network and filled the vein system. Other critical technical specifications for an appropriate contrast agent include:

• Absence of diffusion out of blood vessel after animal death.

• Limited medium shrinkage that could lead to vessel deformation or impaired quantification.

• Addition of inert dye, to provide delineation within the circulatory tree for microscopic vessel examination and analysis.

• Ease of manipulation and injection in small animals.

• Homogeneity of contrast agent solution

Silicone rubber has been extensively used as a filling agent to study micro-vasculature, because of its inert properties. A lead-containing radiopaque silicone rubber called Microfil (Microfil, Flow Tech, Carver, MA) has been widely reported in micro-CT studies[3]. It has a low viscosity that allows it to fill completely the vascular compartments with little resistance[4,5]. Coming from different reports, this compound completely fills the arterial vasculature when perfused at physiological pressure and flows freely from the veins[6,7]. The hydrophobic properties of silicone rubber keep it contained within the vascular compartment, and extravasation has only been reported in situations such as inflammation where physical leaks are present[6].

Neoprene latex 671 (Dupont de Nemours) is a liquid synthetic rubber composed of polychloroprene homopolymer with high tensile strength, high elongation, excellent film formation without curing, and considerable resistance to degradation from chemical or environmental exposure. Because of its low viscosity, this compound fills the arterial vasculature down to 20 μm size vessels when it is perfused at physiological pressure. In our experiments, we never observed any extravasation of latex in tissue or any vessel damage due to neoprene polymerization[8].

Gelatin at 5 to 10% concentration has also been used as a vehicle compound with bismuth or barium sulfate[9,10]. This enabled complete filling of entire microvasculature under physiologically relevant perfusion pressure[11]. Other groups have used Batson's No. 17 polymethylmethacrylate with an added lead pigment[9].

### Contrast agents for in vivo imaging

It is not uncommon for *in vivo *studies to take 30-120 minutes. Such long scan times necessitate either a long-circulating contrast agent or continuous infusion of contrast material. Contrast agents used for *in vivo *imaging can be administered repeatedly to the animal, eliminating the need to sacrifice and assay multiple animals at various time points in longitudinal studies.

Different groups report the use of conventional iodinated vascular contrast agent encapsulated within polyethylene glycol-stabilized liposomes, showing stable enhancement of the intravascular space for more than 3 hr[12]. The Fenestra product line is based on iodinated triglycerides contained in the lipophilic cores of oil-in-water lipid emulsions similar to the naturally-occurring chylomicron remnants. It provides long-lasting visualization of vascular systems with micro-CT. Others have used pluronic F127 and a naturally iodinated compound, Lipiodol to form radiopaque nanoreservoir structures with good success[13].

Collectively, the vasculature can be efficiently explored with a combination of contrast agent and vehicle. Notably, it should be pointed out that the ease of handling is another critical parameter. Investigators could modulate the viscosity of the injected compound depending on the target organ and the vascular bed that require filling.

## Image analysis

**Figure 1:**
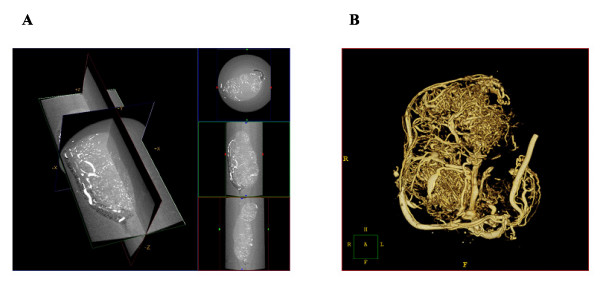
**Micro-CT imaging of a pancreatic tumor vasculature**. Micro-CT images of a pancreatic tumor harvested from the flank of an athymic mouse. The mouse was perfused with Microfil and imaged at 6.5 microns. A. Capillary blood vessels appear in white on the three orthogonal cross-sections of the volume. B) Surface rendering of the micro-CT volume.

**Figure 2:**
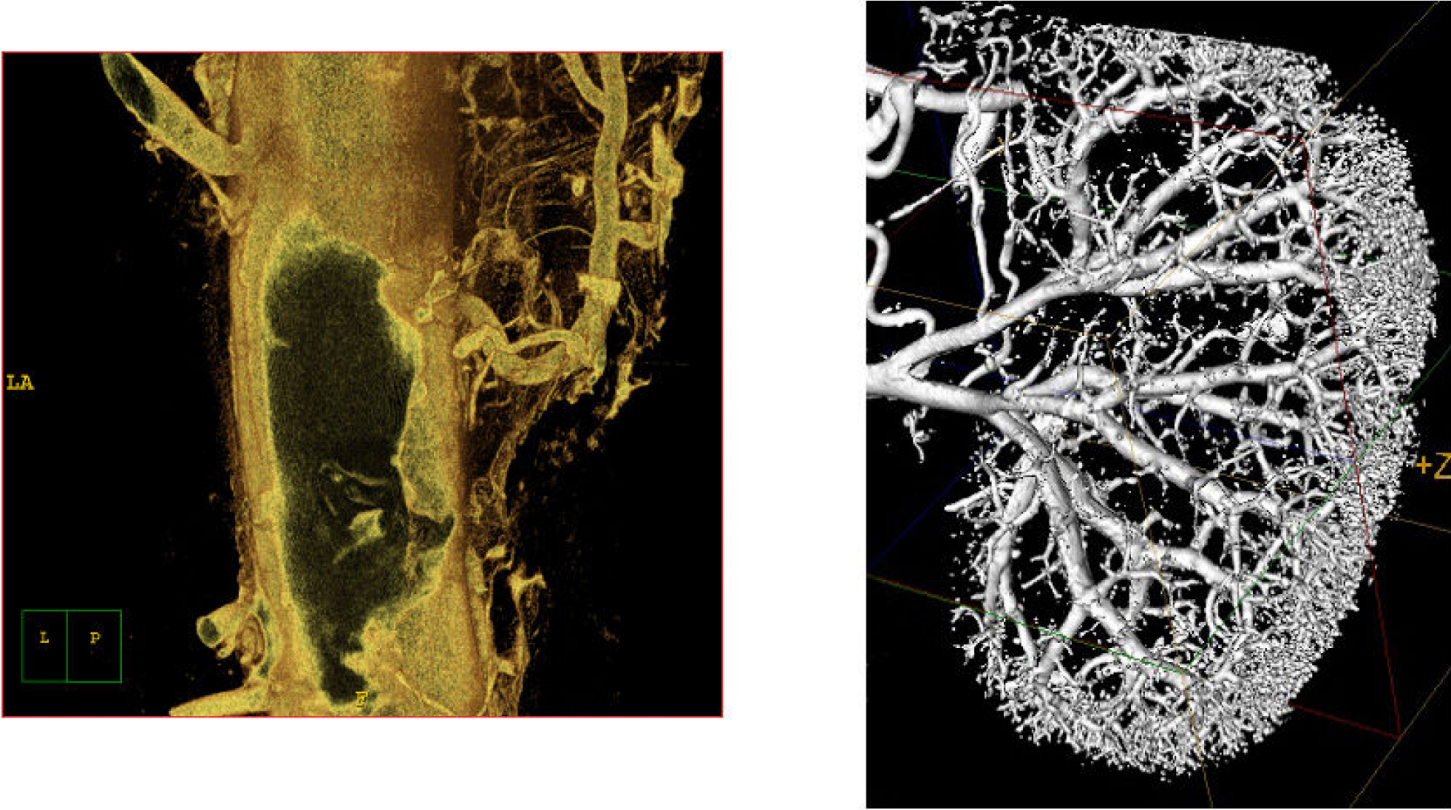
**Examples of different micro-CT applications**: A) atherosclerotic plaque and vasa vasorum in the descending aorta in mice, and B) vascular tree of a mouse kidney.

With the help of contrast agents, micro-CT scanners provide high resolution structural 3D information of blood vessels and offer unique opportunities for imaging, quantification, and analysis of various vascular structures. A number of examples demonstrating the resolution and capabilities of micro-CT are shown in Figures 1 and 2. Image analysis of micro-CT involves techniques such as registration, segmentation, quantification, and visualization.

### Image registration

**Figure 3:**
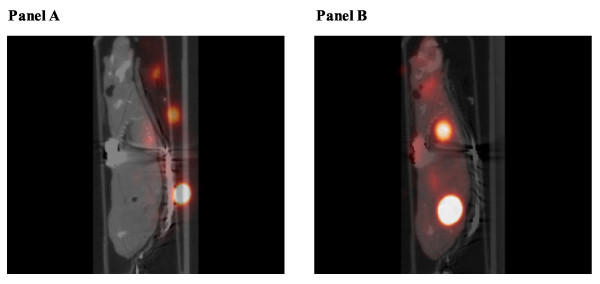
**PET-CT image reconstruction: effect of image registration**. Micro-CT combined with micro-PET before image registration (panel A) and after (Panel B).

Image registration computes a transformation or a deformation field that can be applied to one volume to align with another [14-18]. Specifically, functional imaging modalities such as microPET or SPECT reveal valuable insights into biochemical, physiological, and pharmacological processes in vivo. Their major limitation, however, is the lack of high spatial resolution and detailed anatomical or vascular information. This problem that can be easily alleviated by using complementary information provided by micro-CT and image registration. As illustrated in Figure 3, functional activity can be overlaid with structural information from micro-CT for more detailed analysis and validation of results.

### Segmentation

Segmentation is an image processing technique that partitions an image into meaningful regions or structures of interest. The goal of segmentation is to separate the image points representing a particular object from the rest of the image. Similar to registration, it is another fundamental machine vision task that prepares an image for subsequent quantification and analysis[18]. Among the most common applications of segmentation for micro-CT are studies aimed at anatomical structures, vasculature, and pathologic diseases.

The result of image segmentation is a set of image points representing one or multiple objects. The image points are clustered together based on some common property such as intensity or variation of its rate of change over an image region. Depending on the approach, segmentation could be performed directly on the raw image data or during its visualization and volume rendering. Depending on the user interaction and implementation, it could be either manual or automatic. In manual segmentation, the user interactively traces contours in 2D micro-CT slices to delineate a region of interest and stack the 2D slices in 3D to form the segmented volume. Although this is a very robust approach that works well even with low image quality and little contrast between image structures, it is a tedious and time consuming task better suited for convex or round shaped objects. An automated and robust approach to segmentation is required for more complex objects, such as blood vessels or vascular vessel trees. Thresholding is an image processing technique that tries to separate an object based on its intensity values by setting all image points below and/or above a threshold of certain intensity[18]. It is simple and works well if there is a sharp change of intensity on the boundary of the object and the surrounding image points. It is not very reliable in cases where the object's intensity values merge with the background. One way to increase the contrast of blood vessels in micro-CT is to adjust the concentration of contrast agent. Denser contrast agents result in higher intensity, which differentiates vessels from background, and vice versa. More complex techniques based on partial differential equations modeling physical processes and deformable shapes have also been developed[18]. The best approach depends mainly on the quality of the micro-CT volume. Factors that should be considered are: how well the object of interest stands out from the background, complexity of the structure of interest, reliability and repeatability of the approach.

### Quantification

Since its introduction, software post processing for preclinical micro-CT has posed the widespread acceptance of the need for objective, accurate, and reproducible quantification of results rather than manual measurement.

General techniques for quantification of micro-CT volumes include combinations of morphometric and fractal analysis. Morphometry is the quantitative measurement of structures. Morphometric parameters such as volume, area, connectivity, thickness, and degree of anisotropy are standard for analysis of trabecular bone microstructure[19,20], and were recently applied for quantification of blood vessels[10,21-23]. The morphometric parameters are primarily computed after dimensional reduction of the 3D micro-CT volumes to 2D sections[10,21,23]. Basic morphometric parameters, such as vessel volume, have also been computed directly from the 3D micro-CT volumes[22]. Depending on how the 2D sections are obtained, the resulting morphometric parameters can be used to reconstruct the morphometric parameters in 3D, which provides a 3D interpretation of the planar sections [23] or direct quantitative measures from 2D [10,21]. The measurement of morphometric parameters is relatively simple and is generally carried out as an arithmetical operation over a thresholded binary image that yields information on the properties of the structure of interest.

Fractal analysis has also been used to quantify complex geometric structures in micro-CT volumes[24,25]. A fractal is a quantity that provides a measure of the "self-similarity" of an object at different scales. The more complex the object, the more space it fills. An excellent review of the technique is provided by Baish et al [26]. The calculation of the fractal dimension in micro-CT is straightforward and is performed by thresholding the micro-CT volume to segment out the object of interest and counting the number of voxels representing the object at different scales. A multi-scale fractal analysis of microvascular networks in the brain showed that normal cortical vascular networks have scale-invariant fractal properties on a small local scale as described by Risser et al[27].

#### Quantification of neovasculature with Micro-CT

**Figure 4:**
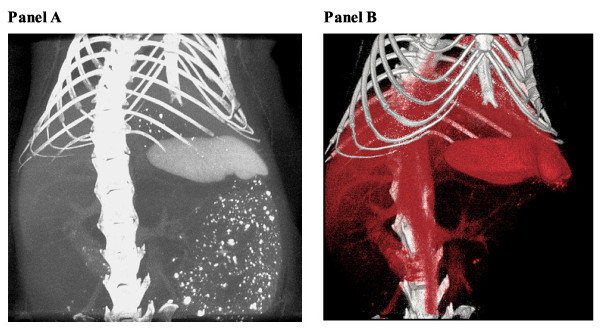
**In vivo micro-CT of a mouse injected with 0.4 mL of Fenestra VC**. A maximum intensity projection is shown in A and its volume rendering in B. The contrast is not sufficinet to visualize small vessels.

Development of therapeutic angiogenesis/arteriogenesis is a frequent goal for ischemic disease research programs, therefore, effort has been put forward in recent years to develop various methods for quantification of neovascularization [21,22,28]. Radiological quantification of angiogenesis/arteriogenesis provides a significant improvement in the tools available for studying and understanding the mechanism of neovascularization. Unfortunately, as illustrated in Figure 4, commercially available *in vivo *contrast agents do not provide enough contrast necessary to visualize small angiogenic vessels, even at the high special resolution of *in vivo *micro-CT scanners. While in vivo experiments are a key goal in vascular imaging, *ex vivo *experiments at present result in much more meaningful data, a consequence of better contrast agents, longer acquisition times, and higher X-ray doses. Specimen micro-CT scanners have a high spatial resolution, which combined with *ex vivo *contrast agents provides a robust methodology for evaluation of intact vascular networks [21,22,29]. Although 3D localization and visualization are important, the power of vascular imaging methods lies in their quantitative analysis, which includes characterization and measurement of different parameters, such as connectivity, anisotropy, thickness distribution, as well as cross-sectional area and volume.

### 2D serial section analysis

Due to large volume size (10^8^-10^10 ^voxels), processing, transmitting, and archiving of micro-CT data as well as its intelligent analysis within a reasonable time frame is a real challenge. An efficient 2D serial section analysis method was initially proposed by Li W, et al[21]. A global threshold to visualize 2D vasculature and eliminate bone using a modified Image Pro-Plus 5.0 algorithm (Media Cybernetics, Silver Spring, MD) was applied to 500 serial slices from the upper limb and 250 serial slices from the lower limb to segment and expressed the data as vascular segment number and volume. The algorithm was built within the AutoPro programming language (Visual Basic SAX engine) inside Image-Pro Plus. They summed up serial 2D imaging information to the 3D volume.

**Figure 5:**
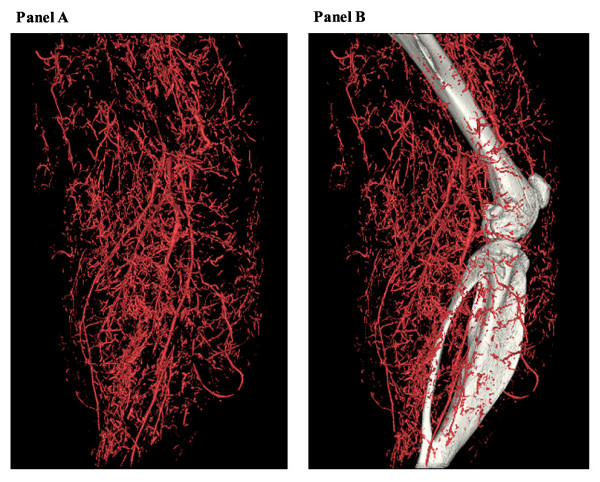
**High-resolution micro-CT 3D volume rendering of ligated hindlimb filled by 30% bismuth**. A. Vasculature has been extracted from the raw data at 24 μm resolution. There is trade-off between the field of view and the maximal resolution. B. Vascular tree has been overlapped by the skeleton system, which demonstrates the 3D relationship between them.

As illustrated in Figure 5, Zhuang and colleagues have successfully established the dynamic 3D geometry of the entire peripheral arterial tree in a mouse hindlimb[28]. The major focus now is to develop an automated algorithm to extract detailed morphometric data such as the diameters, area, number of vessels, and distributions of different size of vessels. Ideally, such methods for vascular analysis of micro-CT would involve five principle steps: 1) segmentation of arteries from bone and contaminated venous system; 2) re-creation of the 3D vascular tree according to a re-orientated central line along the long axis of the major vessels (femoral or anterior tibial artery); 3) evaluation of the mean diameter and cross-sectional area of the segmented branches in each slice; 4) estimation of the distribution of the diameter of blood vessels into different groups; and 5) computation of the total area and volume in the serial slices of both upper and lower limbs.

### 3D-based quantification

Quantitative methods for cancellous bone architecture have been used to evaluate the histomorphometric values of vascular networks[22]. These parameters include vessel volume, connectivity, number, thickness, thickness distribution, separation, and degree of antisotropy. Using this method, vessel volume can be computed based on the voxel size and the number of segmented voxels in the 3D image after application of the binarization threshold. It is really challenging to define the universal binarization threshold for all evaluations within a study, which is highly dependent on the scanning resolution, imaging quality, and decalcification effect. If a value of the binarization threshold is set too high, smaller vessels will be erased from the image. In that case, the volume will be down-estimated. If a threshold is too low, vessels will appear artifactually large and the volume will be over-estimated.

Connectivity is defined as the maximal number of branches that may be cut without separating the structure. Connectivity can easily be calculated from a 3D dataset by using the Euler number[30]. As pointed out, the edge problem needs special attention in all quantifications of connection and adjustment of the Euler number might be necessary[30].

Vessel number, thickness (lumen diameter), thickness distribution, and separation can be calculated using a model-independent method for assessing thickness in 3D volumes[8,22,31]. This technique defines a local thickness at every voxel in the volume of interest (VOI) as the diameter of the largest sphere that both contains the voxel and is completely within the structure of interest. The average of the local voxel thicknesses yields the vessel thickness parameter and a similar calculation on the background voxels determines the vessel separation. To calculate vessel number, the segmented volume is skeletonized, leaving just the voxels at the mid-axes of vessels in the structure. Vessel number is defined as the inverse of the mean spacing between the mid-axes of the structures in the segmented volume.

Anisotropy describes the degree to which the direction of the segmented 3D vascular bed is oriented. It can be determined by the mean intercept length, the volume orientation, star volume and length distribution[30]. Duvall CL, et al [22] used *eigen *analysis to find the principal material directions from a local image neighborhood and determined the eigenvalues (ratio of the maximum and minimum radii of the ellipsoid) as described by Gundersen et al. [32]. It can be done at the scale of a group of vessels. A degree of anisotropy of 1.0 denotes that the vascular network is perfectly isotropic, and higher values of degree of anisotropy indicate that a structure contains a preferential material direction. In a recent contribution by Risser et al[33], anisotropy has been used to merge discontinuities in 3D images of vascular structures representing undesirable gaps in vascular networks.

Although 3D data has a dimensional advantage over its 2D counterpart, there is very little, if any, commercially available software for real 3D quantification of blood vessels from micro-CT. Novel quantitative methods and clear definitions of what needs to be quantified are yet to be developed. What we do know is that most of the currently available software tools are for quantification of bone, and before we use them for vascular exploration, we have to determine the extent to which the neovasculature is similar to the trabecular network of rod-like and plate-like structures.

## Limitations of micro-CT in vascular research

### Analysis and quantification

Micro-CT scanners that provide a resolution between 1 and 100 μm can be used for vascular research. If the imaging targets are small capillaries, scanners close to the 1 μm limit provide a significant advantage. Regardless of the resolution, blood vessels in Micro-CT volumes cannot be differentiated from soft tissue without a contrast agent. Consequently, the use of the imaging modality is limited by the properties of the contrast agent (e.g. concentration, viscosity, opacity, etc.). A poorly chosen contrast agent will result in a low image quality that can be very difficult to analyze or quantify.

The main weakness of micro-CT for vascular research, however, is the lack of appropriate software tools for image analysis and quantification. This is partially due to the tortuous nature of blood vessels. For example, peripheral vasculature consists of a combination of tree and arcade patterns. After ischemia, angiogenesis and arteriogenesis form other patterns such as anastomoses and collaterals, which make the vascular tree even more complicated. In order to quantify collateral growth, scientists must be able to differentiate existing from newly grown vessels in 3D. This is not a trivial image processing task and new algorithms must be developed. Commercially available morphometric analysis tools for bone applications have found wide acceptance in the vascular community due to the lack of better alternatives. Those tools perform 2-D serial slice analysis of micro-CT along a given axis[10,19-21,23]. When applied to vascular micro-CT volumes, such tools usually output the number of blood vessels with a given diameter and volume. These results are conceptually inaccurate, because they depend upon the orientation of the sample and carry an unknown amount of error caused by the dimensional reduction. Depending on the orientation of the sample, estimation of vessel diameter from 2-D may not be possible, because vessels do not always appear as circles on planar sections. Such limitations raise serious questions about the accuracy of serial 2-D slice analysis when applied to vascular micro-CT volumes.

### Blood flow

Despite the recent development of morphometric data in the peripheral vasculature and hence the sophistication of the resulting hindlimb models, a rigorous hemodynamic analysis of spatial perfusion of the skeleton muscle is still unattainable. The reason for the inaccessibility of such a model is the lack of quantitative data on the spatial relation between the skeleton muscle and the supplying vessels. Therefore, methods for reconstruction of the 3D branching pattern of the peripheral vessels for a spatial analysis of hindlimb blood flow are also very important.

### Micro-CT and histology

**Figure 6:**
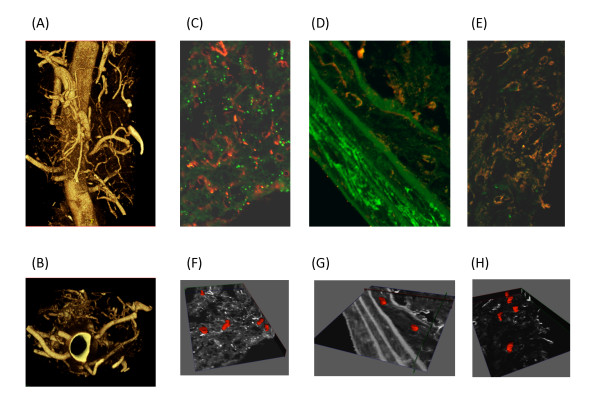
**Imaging the vasa vasorum**. Micro CT and confocal microscopy were used to image the vasa vasorum (A) Mice were anesthetized, heparinized and perfused. A silicone rubber compound, Microfil Blue, was infused through the aortic cannula. Descending aortas with a wide adventitial margin were removed after polymerization was complete, then scanned with a GE eXplore Locus SP microCT scanner at 6.5 micron resolution. Three-dimensional volumetric images were reconstructed from acquired two dimensional projections without averaging, yielding a final voxel size of 6.5 microns (B) Reconstructed images were rotated to obtain luminal images from mice. Descending aorta cross sections probed for smooth muscle actin (green) and lectin (red) were imaged by confocal microscopy (C) adventitia, (D) vessel wall, (E) plaque. To visualize blood vessels in the (F) adventitia, (G) vessel wall and (H) plaque the reconstructed Z-stacks were manually segmented to represent co-localized probes in consecutive axial slices. The obtained contours were modeled and stacked in 3-D for volumetric surface representation.

Correlation of micro-CT data with optical microscopy may help in interpretation of biological results by using complementary information from the two imaging modalities. However, it is not always possible to perform microscopy on sections obtained from ex vivo samples previously imaged with micro-CT and correlate results. In addition, more careful inspection of micro-CT sections imaged with a confocal microscope also indicates that not all blood vessels get filled with contrast (Figure 6). Depending on the type of contrast, the size of its particles, viscosity, and properties, different level of detail in micro-CT are obtained. A quantitative measure that would indicate the amount of difference in blood vessel volume from micro CT and microscopy is needed.

## Other vascular applications and uses of micro-CT

Neo-angiogenesis associated with more advanced stages of human atherosclerosis is found in plaque and the vasa vasorum[34]. Studies utilizing micro-CT and immunohistochemistry techniques demonstrate that the presence and extent of vasa vasorum correlate with atherosclerotic lesion size and lumen diameter in hypercholesterolemic animal models[35]; inhibition of neovascularization in the vasa vasorum is associated with reduced plaque progression[36].

Advancements in micro-CT technology and image reconstruction software have enabled more in depth exploration of the vasa vasorum to result in identification of anatomically different types of vasa vasorum and their origin, measurement of density, branching pattern and blood flow through the vasa vasorum (Figure 6)[4,37]. These advancements provide a means of examining the role of the vasa vasorum in the context of atherosclerosis, abdominal aortic aneurysm, restenosis following angioplasty, metabolic syndrome, type 2 diabetes mellitus as well as studying the effects of drug-eluting stents [38].

Calcification or hemorrhage within the arterial wall during atherosclerosis process or tissue damage have been accurately assessed by micro-CT[39,40]. Basic micro-architectural structures of lungs and pulmonary-vascular system have been successfully visualized and quantitated in small rodent by micro-CT[41].

## Perspective in micro-CT improvement

Single-energy micro-CT is still limited in cases where two different materials share similar grey-scale intensity values. Contrast among soft tissue components could be increased using the implementation of dual energy x-ray subtraction algorithms. X-ray diffraction technique, wide angle X-ray scattering, refraction-based imaging could also increase the contrast[40,42]. Highest resolution with voxel < 1 μm^3 ^imaging cellular or sub-cellular feature can be achieved with synchrotron radiation-based scanners (high energy and narrow bandwidth). The use of multi x-ray source/detector array scanners can increase scan speed for application to living animals[40].

## Conclusion

Micro-CT is one of the most promising imaging modalities for vascular exploration. Its extremely high spatial resolution presents a unique opportunity for studying the structure, organization, and, to some extent, even the function of blood vessels. The imaging process is not straightforward and spans over a number of different areas ranging from animal handling and preparation to image processing and quantification. Successful Micro-CT imaging requires perfection at each step. Better contrast agents and delivery vehicles are needed for more detailed exploration of small angiogenic vessels. Robust image processing techniques that can facilitate accurate quantification of micro-CT data in 3D have to be developed. Correlation of microscopy with micro-CT for interpretation of results is also very important.

## Competing interests

The authors declare that they have no competing interests.

## Authors' contributions

LZ carried out mCT image analysis, and wrote the "image analysis" and "limitations" sections. PO carried out the experiments comparing different animal preparations and contrast agents. ZWZ carried out most of the animal experiments. KM was expert in animal preparation and wrote the "animal preparation " section. MJMK carried out the experiments on vasa-vasorum and histological analysis and proofread the manuscript. MS and TC conceived of the study and coordinated the writing.

All authors read and approved the final manuscript

## Supplementary Material

Additional file 1 **Contrast agents for ex vivo and in vivo imaging**. this paragraph gives more details on contrast agents properties and use than the section in the manuscript. Click here for file
