# Supplementary material for: Micro computed tomography for vascular exploration
Source: J Angiogenes Res. 2010 Mar 5;2:7. doi: 10.1186/2040-2384-2-7 (PMC2841094; doi:10.1186/2040-2384-2-7)
Supplement: Additional file 1 — Contrast agents for ex vivo and in vivo imaging. this paragraph gives more details on contrast agents properties and use than the section in the manuscript. [file 2040-2384-2-7-S1.DOC]

# Additional files

**Additional file 1**

**Title: contrast agents for ex vivo and in vivo imaging**

**Description: this paragraph gives more details on contrast agents properties and use than the section in the manuscript.**

**Contrast Agents**

Unlike bony anatomy, blood vessels provide very little inherent contrast for micro-CT imaging. The implementation of novel vascular contrast agents and vehicle has resulted in several new applications for micro-CT in the evaluation of micro vascular anatomy. Important functional and technical specifications of such contrast agents include high radio opacity, ease of manipulation and injection and physical properties adaptable to filling the desired vascular bed or organ.

Most clinical X-ray contrast agents are based on the element iodine. They are water soluble and therefore suitable for clinical radiography applications. Radiological contrast agents are water soluble solutions as well. One commonly used variety is based on a suspension of large insoluble particles of barium sulphate. The latter have better coating properties than the iodinated contrast media, and tend to form thin layers spread over the lining of tubes. However, it has been proven that insoluble particles tend to settle quickly and impair contrast homogeneity. This problem could be solved in part by using pulverized barium sulphate with 1μm particles. Many other elements have higher atomic numbers than barium and can be used as contrast agents. Bismuth and lead (Pb) are good examples.

However, not only the type of contrast agent, but also the delivery vehicle, the protocol of injection, and the micro-CT acquisition may influence the end results. It has been reported that the contrast in individual vessels ranges from 60 – 1500 HU for a corrosion casting material with an added lead pigment, from 2500 – 5000 for silicon rubber with lead pigment, and from 7000 – 11 000 HU for a suspension of barium sulfate in gelatin[1].

***Contrast agents for ex vivo imaging***

Viscosity is one of the most important properties of the vehicle because it influences contrast agent behavior and its applications for micro-CT. The viscosity of a liquid is assessed by measuring its rate of flow through a standard thin capillary tube under standard conditions of pressure and temperature. The practical importance of viscosity of a contrast medium relates mainly to the force that is required to inject it through a needle into the entire vascular bed, which limits the quantity and the pressure at which it can be injected. High injection pressures may lead to rupture of vascular beds.

Since viscosity is inversely related to temperature, warming the contrast medium may partly resolve this problem, but it introduces the inconvenience of immediate use of the contrast medium [2, 3].

The contrast agent must completely fill the vascular network to allow a precise study of the microcirculation: the actual resolution of vessels imaged with micro-CT is in the range of 5 to 20 μm (i.e., the effective voxel size). Depending on the contrast properties (especially viscosity), arterial and/or venous system can be injected. An increased viscosity limits vehicle penetration at the arteriolar level only. This aspect could be a great interest in arterial / venous delineation in micro-CT analysis. Other critical technical specifications for an appropriate contrast agent include:

- Absence of diffusion out of blood vessel after animal death.
- Limited medium shrinkage that could lead to vessel deformation or impaired quantification.
- Addition of inert dye, to provide delineation within the circulatory tree for microscopic vessel examination and analysis.
- Ease of manipulation and injection in small animals.
- Homogeneity of contrast agent solution

Because of its inert properties, silicone rubber has been extensively used as a filling agent to study micro-vasculature. A lead-containing radiopaque silicone rubber called Microfil (Microfil, Flow Tech, Carver, MA) has been widely reported in micro-CT studies. It has a low viscosity that allows it to fill completely the vascular compartments with little resistance. It polymerizes at room temperature with minimal shrinkage, allowing quantitative measurements. To achieve a viscosity level suitable for injection of the microcirculation, it is necessary to blend the compound with an equal quantity of diluent. Viscosity ranges from 20 to 30 centipoise. Working time is 20 minutes and begins with the addition of curing agent. Coming from different reports, this compound completely fills the arterial vasculature when perfused at physiological pressure and flows freely from the veins [4-8]. The hydrophobic properties of silicone rubber keep it contained within the vascular compartment, and extravasation has only been reported in situations such as inflammation where physical leaks are present[4].

Neoprene latex 671 (Dupont de Nemours) is a liquid synthetic rubber composed of polychloroprene homopolymer with high tensile strength, high elongation and excellent film formation without curing and considerable resistance to degradation from chemical or environmental exposure. Initial pH is 12.5, and neoprene polymerizes when in contact with pH 8–9. Viscosity is 50 centipoise. Because of its low viscosity, this compound fills the arterial vasculature down to 20μm size vessels when they are perfused at physiological pressure. We could point out that this neoprene solution never fills the venous system when it is injected in the arterial network. In our experiments, we never observed any extravasation of latex in tissue or any vessel damage due to neoprene polymerization.

Gelatin at 5 to 10% concentration has also been used as a vehicle compound with bismuth or barium sulfate. Warmed medium (42°C) was injected in the vasculature and the animals were immediately chilled on ice to allow solidification of gelatin within 30 s throughout the organism [1, 9]. This enabled complete filling of entire microvasculature under physiologically relevant perfusion pressure[10]. Other groups have used Batson’s No. 17 polymethylmethacrylate with an added lead pigment[1].

***Contrast agents for in vivo imaging***

It is not uncommon for *in vivo* studies to take 30-120 minutes. Such long scan times necessitate either a long-circulating contrast agent or continuous infusion of contrast material. Contrast agents used for *in vivo* imaging can be administered repeatedly to the animal, eliminating the need to sacrifice and assay multiple animals at various time points in longitudinal studies.

Different groups report the use of conventional iodinated vascular contrast agent encapsulated within polyethylene glycol–stabilized liposomes, showing stable enhancement of the intravascular space for more than 3 hr [11]. The Fenestra product line is based on iodinated triglycerides contained in the lipophilic cores of oil-in-water lipid emulsions similar to the naturally-occurring chylomicron remnants. It provides long-lasting visualization of vascular systems with micro-CT. Others have used pluronic F127 and a naturally iodinated compound, Lipiodol to form radiopaque nanoreservoir structures with good success[12]. These agents give good contrast discrimination between the myocardium and cardiac blood pool, and fill large and small vessels with high contrast. The properties of these agents, as shown in Table S1, fulfill the imaging requirements of modern preclinical studies. Moreover, the degree of iodine leakage, the risk of renal toxicity as well as anaphylaxis are low.

Collectively, the vasculature can be efficiently explored with a combination of contrast agent and vehicle. Notably, it should be pointed out that the ease of handling is another critical parameter. Investigators could modulate the viscosity of the injected compound depending of the target organ and the vascular bed to fill and study.

**Table S**1: Currently available contrast agents.

| Agent | Temperature | Viscosity | Time to cure | Type | Histology | Filling degree |
| --- | --- | --- | --- | --- | --- | --- |
| Microfil© | ambient | 20-30 | 20 min | Ex Vivo | ++ | Arteries and veins |
| Latex | ambient | 50 | 10 sec | Ex Vivo | ++ | Arteries |
| Gelatin | 42°C | 30-50 | 30 sec | Ex Vivo | +/- | Arteries and veins |
| Iodinated liposomes  Fenestra© | ambient | 15-20 | N/A | In Vivo | N/A | Arteries and veins |

1. Marxen M, Thornton MM, Chiarot CB, Klement G, Koprivnikar J, Sled JG, Henkelman RM: **MicroCT scanner performance and considerations for vascular specimen imaging.** *Med Phys* 2004, **31:**305-313.
2. Plouraboué F, Cloetens P, Fonta C, Steyer A, Lauwers F, Marc-Vergnes JP: **X-ray high-resolution vascular network imaging**. *J Microsc* 2004, **215**: 139-148.
3. Loo BW, Sauerwald IM, Hitchcock AP, Rothman SS: **A new sample preparation method for biological soft X-ray microscopy: nitrogen-based contrast and radiation tolerance properties of glycol methacrylate-embedded and sectioned tissue**. *J. Microsc* 2001, **204**: 69 -86.
4. Bentley MD, Ortiz MC, Ritman EL, Romero JC: **The use of microcomputed tomography to study microvasculature in small rodents.** *Am J Physiol Regul Integr Comp Physiol* 2002, **282:**R1267-1279.
5. Cheema AN, Hong T, Nili N, Segev A, Moffat JG, Lipson KE, Howlett AR, Holdsworth DW, Cole MJ, Qiang B: **Adventitial microvessel formation after coronary stenting and the effects of SU11218, a tyrosine kinase inhibitor.** *J Am Coll Cardiol* 2006, **47:**1067-1075.
6. Kwon HM, Sangiorgi G, Ritman EL, Lerman A, McKenna C, Virmani R, Edwards WD, Holmes DR, Schwartz RS: **Adventitial vasa vasorum in balloon-injured coronary arteries: visualization and quantitation by a microscopic three-dimensional computed tomography technique.** *J Am Coll Cardiol* 1998, **32:**2072-2079.
7. Kwon HM, Sangiorgi G, Ritman EL, McKenna C, Holmes DR, Jr., Schwartz RS, Lerman A: **Enhanced coronary vasa vasorum neovascularization in experimental hypercholesterolemia.** *J Clin Invest* 1998, **101:**1551-1556.
8. Langheinrich AC, Leithauser B, Rau WS, Bohle RM: **[Cardio-pulmonary vascular system. Three-dimensional quantitative evaluation by microcomputed tomography].** *Pathologe* 2004, **25:**135-140.
9. Tirziu D, Moodie KL, Zhuang ZW, Singer K, Helisch A, Dunn JF, Li W, Singh J, Simons M: **Delayed arteriogenesis in hypercholesterolemic mice.** *Circulation* 2005, **112:**2501-2509.
10. Toyota E, Fujimoto K, Ogasawara Y, Kajita T, Shigeto F, Matsumoto T, Goto M, Kajiya F: **Dynamic changes in three-dimensional architecture and vascular volume of transmural coronary microvasculature between diastolic- and systolic-arrested rat hearts.** *Circulation* 2002, **105:**621-626.
11. Mukundan S, Jr., Ghaghada KB, Badea CT, Kao CY, Hedlund LW, Provenzale JM, Johnson GA, Chen E, Bellamkonda RV, Annapragada A: **A liposomal nanoscale contrast agent for preclinical CT in mice.** *AJR Am J Roentgenol* 2006, **186:**300-307.
12. Ho Kong W, Jae Lee W, Yun Cui Z, Hyun Bae K, Gwan Park T, Hoon Kim J, Park K, Won Seo S: **Nanoparticulate carrier containing water-insoluble iodinated oil as a multifunctional contrast agent for computed tomography imaging.** *Biomaterials* 2007, **28:**5555-5561.
